# Citrus Peels in Health Foods: A Case Study of Pulp-Free Japanese-Grown Bushukan (*Citrus medica* var. *sarcodactylis*)

**DOI:** 10.3390/metabo16040254

**Published:** 2026-04-10

**Authors:** Jun Nakahigashi, Eiji Kobayashi

**Affiliations:** 1 Wellness Development Research Center, AIR WATER INC., 1-7, Tsukisamu Higashi 2-jo 16-chome, Toyohi-ra-ku, Sapporo 062-0052, Hokkaido, Japan; nakahigashi-jun@awi.co.jp; 2 Kobayashi Regenerative Research Institute, LLC, 1 Chayano-cho, Wakayama-shi 640-8263, Wakayama, Japan

**Keywords:** *Citrus medica* var. *sarcodactylis*, hesperidin, nobiletin, citrus peel-derived materials, flavonoid quantification, drying methods, HPLC, functional raw material

## Abstract

**Highlights:**

**What are the main findings?**

- Japanese-grown bushukan (*Citrus medica* var. *sarcodactylis*) contains exceptionally low or undetectable levels of hesperidin and nobiletin.
- The hesperidin concentration in both freeze-dried and hot-air-dried samples was approximately one hundredth of that typically found in early-season satsuma mandarin peel.

**What are the implications of the main findings?**

- Functional value should not be presumed based solely on botanical classification, as secondary metabolite profiles can differ markedly even within the same genus.
- The study underscores the necessity of species- and origin-specific compositional verification prior to utilizing citrus peels as raw materials for health foods.

**Abstract:**

**Background/Objectives:** Citrus peels are widely utilized as functional ingredients in health foods; however, their functional value is often assumed based on botanical classification rather than verified chemical composition. Bushukan (*Citrus medica* var. *sarcodactylis*) was selected as it lacks developed edible pulp; consequently, the usable portion consists almost entirely of peel tissue, making it a suitable model for evaluating peel-specific functional components. This commentary highlights the importance of species- and origin-specific evaluation through a case study of Bushukan (*Citrus medica* var. *sarcodactylis*) whole fruit powder cultivated in Japan. **Methods:** Dried whole-fruit powder samples of bushukan, prepared by freeze-drying and hot-air drying at 50 °C, were analyzed, and the contents of hesperidin and nobiletin were quantified using high-performance liquid chromatography (HPLC) following methanol reflux extraction. **Results:** Hesperidin was detected at 75 mg/100 g under both drying conditions, whereas nobiletin was below the practical limit of quantification (approximately 1 mg/100 g). No reduction in hesperidin content was observed after drying at 50 °C. These levels were markedly lower than those reported for commonly used citrus peels, such as satsuma mandarin, in previous studies. **Conclusions:** This commentary demonstrates that Japanese-grown bushukan samples do not necessarily provide substantial levels of commonly expected citrus flavonoids. These findings underscore the need for species- and origin-specific compositional verification before the use of citrus peels as raw materials for health food applications, illustrating this need through a practical, cautionary case study.

## 1. Introduction

Citrus peels have been widely used as ingredients in health foods because of their richness in flavonoids and other bioactive compounds with reported biological activities. Such applications have been particularly explored in East Asian food and nutraceutical contexts. However, the composition of citrus peels varies markedly depending on species, origin, and production context, and functional equivalence should not be assumed.

*Citrus medica* var. *sarcodactylis* is an evergreen shrub in the family Rutaceae, genus *Citrus*, and is classified as a sour/acidic citrus variety similar to “kabosu” and “yuzu”. The fruit is highly fragrant, ripens to a deep yellow, and exhibits an elongated morphology with an apex divided into finger-like segments. Owing to its hand-shaped appearance suggestive of joined palms, the fruit is affectionately called “Buddha’s hand” or “fingered citron” [1,2].

Peels from the genus *Citrus* are rich in health-promoting constituents and have been valued since antiquity. We previously reported that two peel-derived flavonoids, hesperidin and nobiletin, alleviate adverse behaviors in senior dogs, such as fear- and anxiety-induced howling [3]. Building on these findings, a peel-based product for companion senior dogs was formulated, and the absence of adverse events was demonstrated in a high-dose, repeated-administration study [4]. Critical to these applications is the observation that citrus peels do not exert uniform health effects across varieties; therefore, routine target-compound analysis is indispensable. This article reports a targeted evaluation of two flavonoids, hesperidin and nobiletin, in dried, powdered Buddha’s hand samples.

*Citrus medica* var. *sarcodactylis* is primarily cultivated in China for use in traditional Chinese medicine [5], although cultivation also exists in Japan, particularly in the Kagoshima and Wakayama Prefectures. Buddha’s hand fruits produced in Wakayama (Kobayashi Regeneration Institute LLC, Wakayama) were analyzed in this study, with samples prepared using two distinct drying approaches.

When citrus peels are intended for food use, their chemical composition should be evaluated with careful consideration of species, cultivation area, and harvesting conditions, as these factors can substantially influence component profiles. In this context, bushukan was selected as a suitable case material because it lacks a developed edible pulp; consequently, the usable portion consists almost entirely of peel tissue, and the entire fruit was dried, pulverized, and analyzed to evaluate peel-specific functional components. Citrus peel-derived materials are frequently incorporated into health foods based on presumed functional equivalence, highlighting the importance of verifying their actual chemical composition prior to use.

Bushukan (*Citrus medica* var. *sarcodactylis*) was selected for this study because it lacks developed edible pulp, and the usable portion consists almost entirely of peel tissue, making it a suitable model for evaluating peel-specific functional components.

Citrus peels are widely used in health foods due to their richness in flavonoids and other bioactive compounds with reported biological activities.

## 2. Materials and Methods

Hesperidin (purity ≥ 98.5%) and nobiletin (purity ≥ 98%) standards were purchased from Funakoshi Co., Ltd. (Tokyo, Japan). Liquid chromatography–mass spectroscopy-grade methanol (Kanto Chemical Co., Inc., Tokyo, Japan) was used throughout this study. Components were analyzed using high-performance liquid chromatography (HPLC) to quantify hesperidin and nobiletin concentrations in the powder.

A 200 mg portion of sample powder was accurately weighed and extracted with 10 mL of methanol by refluxing at 80 °C for 1 h. The supernatant was transferred to a 20 mL volumetric flask. The residue was re-extracted with 10 mL of methanol under identical conditions, and the combined extracts were adjusted to a final volume of 20 mL [6].

A 2 μL aliquot of this solution was filtered, diluted as necessary, and injected into an HPLC system (Agilent 1200 Series, Agilent Technologies, Inc., Santa Clara, CA, USA) equipped with a Fusion C30 analytical column (2.0 × 100 mm, 3 μm; Nomura Chemical Co., Ltd., Seto City, Japan). The mobile phase comprised 10 mM aqueous phosphoric acid and methanol at a flow rate of 0.5 mL/min. The gradient program for hesperidin included an increase from 25% methanol to 40% over 0–15 min, then to 40–95% at 15–16 min, followed by a 4 min hold at 95% methanol. For nobiletin, the gradient was initiated at 50% methanol, increased from 50% to 95% over 0–15 min, followed by a 5 min hold. The column temperature was maintained at 40 °C, and detection was conducted at 285 nm. A methanol solution prepared under identical conditions without the sample served as the blank. All samples were analyzed in duplicate at the sample preparation level, while repeatability and low-concentration measurements were evaluated using six replicate injections.

The HPLC method used in this study was based on an established assay routinely applied for citrus flavonoid analysis [6]. Linearity was confirmed using external calibration curves prepared from authentic standards of hesperidin (1–10 μg/mL) and nobiletin (0.2–2 μg/mL), with correlation coefficients exceeding 0.999 for both analytes.

Repeatability was assessed using six replicate injections of standard solutions (hesperidin: 5 μg/mL; nobiletin: 1 μg/mL), yielding relative standard deviation (RSD) values of 1.3% and 0.94%, respectively. The limits of detection (LODs) and quantification (LOQs) were estimated based on six replicate measurements of a low-concentration standard solution (0.1 μg/mL), using three and ten times the standard deviation of the observed signal, respectively. These evaluations were conducted to confirm the suitability of the method for descriptive compositional analysis in the context of this case study.

## 3. Results

Hesperidin was detected at 75 mg/100 g in both the 50 °C and freeze-dried samples, which is approximately one-hundredth of that typically observed in early-season satsuma mandarin peel powder (8.5–9.3 g/100 g). Furthermore, the nobiletin levels in the samples subjected to both drying treatments were below the practical limit of quantification (approximately 1 mg/100 g) (early-season satsuma mandarin peel: 41–47 mg/100 g). No reduction in hesperidin content was observed after drying at 50 °C. Owing to the resulting lower moisture content, this material is easily processed and serves as a technically processable raw material, although it is not suitable as a source of the targeted flavonoids.

Based on replicate measurements of a 0.1 μg/mL standard solution, the estimated LOD and LOQ values were 0.156 and 0.204 μg/mL for hesperidin and 0.028 and 0.109 μg/mL for nobiletin, respectively. Given the calibration ranges employed in this study (hesperidin: 1–10 μg/mL; nobiletin: 0.2–2 μg/mL), quantification below 1 μg/mL should be regarded as descriptive rather than fully quantitative. Accordingly, concentrations of nobiletin in bushukan samples were considered practically non-quantifiable under the present analytical conditions.

**Figure 1:**
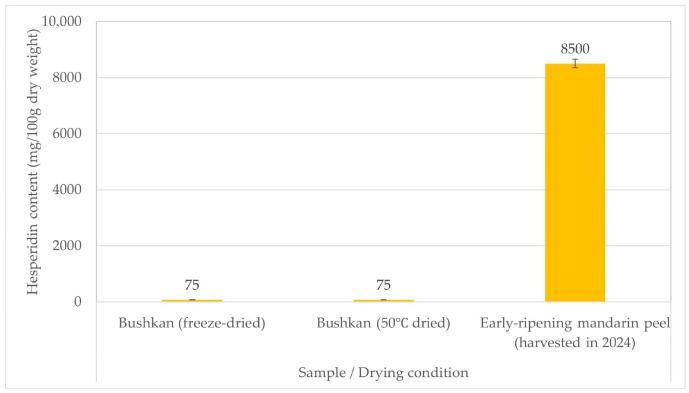
Comparison of hesperidin content in Japanese-grown bushukan samples under different drying conditions (freeze-dried and hot-air dried at 50 °C), in relation to literature values reported for early-season satsuma mandarin peel powder. Values represent mean values derived from duplicate measurements. Nobiletin levels were below the practical limit of quantification under the present analytical conditions.

**Figure 2:**
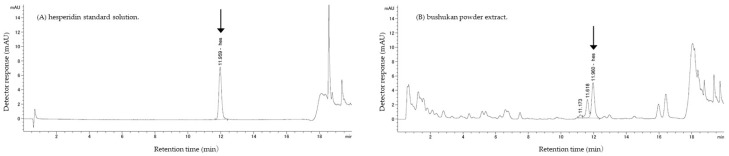
Representative HPLC chromatograms of (**A**) hesperidin standard solution and (**B**) bushukan powder extract. The retention time corresponding to hesperidin is indicated by an arrow. Nobiletin was not observed at a quantifiable level under the present analytical conditions.

Representative HPLC chromatograms and a comparative summary of flavonoid levels are shown in Figure 1 and Figure 2.

The LOD and LOQ values expressed in μg/mL refer to the concentration in the injected solution, whereas values expressed in mg/100 g refer to the corresponding concentrations calculated for the powdered samples.

## 4. Discussion

Even within the genus Citrus, secondary metabolite profiles differ substantially among species and cultivars. The low hesperidin levels observed in *Citrus medica* var. *sarcodactylis* may reflect these species-specific metabolic characteristics. Because all samples were collected at commercial maturity, the potential effects of fruit developmental stage on flavonoid content could not be evaluated and represent a limitation of this study.

Recent investigations have discovered new limonoid compounds, with studies characterizing their chemical structures and effects on suppressing fat accumulation [7]. Additionally, because bushukan is also used as a fragrance material, it is necessary to determine whether it contains other functional constituents, including volatile compounds [1]. Moreover, analysis of post-juicing peel from U.S.-origin Valencia oranges revealed hesperidin and nobiletin concentrations of 3.6 g/100 g and 38 mg/100 g, respectively. The Valencia orange is a cultivar of sweet orange (*Citrus sinensis* (L.) Osbeck), which, similar to Buddha’s hand, belongs to the family Rutaceae and the genus *Citrus* [8] and is widely used for processing, particularly as a raw material for juice because of its high juice content. Even within the same family (Rutaceae) and genus (*Citrus*), the types and contents of components differ; therefore, component analysis remains critical. Furthermore, component levels fluctuate according to the harvest year and season [3].

Although agricultural peels contain abundant useful components such as polyphenols [9], comprehensive analyses are required to address the specific challenges associated with agricultural products for commercial utilization. When utilizing dried peel-derived materials, compliance with residual pesticide standards in the fresh state alone is insufficient; residual pesticide analysis must be conducted post-drying, as components become concentrated during the drying process. Moreover, the risk of “spray drift,” defined as the unintended dispersal of pesticides from adjacent areas, requires caution even for crops cultivated without direct pesticide application [10,11].

The present analysis is not intended to generalize functional properties across citrus species but rather to demonstrate how peel composition can differ markedly depending on species and production context, thereby underscoring the necessity of case-by-case evaluation for food applications. This suggests that components other than flavonoids, such as volatile aromatic compounds or limonoids, may represent more characteristic functional constituents of bushukan samples.

In parallel with such case-by-case compositional evaluation, we are extending these compositional assessments to peels from late-season citrus varieties and related citrus species. By systematically assessing peel-derived components in relation to both efficacy and safety for specific target conditions, we aim to support the rational use of citrus peels as functional raw materials while maintaining consistency in biological effects and safety profiles.

## 5. Conclusions

This commentary presents a case study demonstrating that Japanese-grown bushukan samples contain low or undetectable levels of two widely expected citrus flavonoids: hesperidin and nobiletin. Rather than identifying novel compounds, the purpose of this work is to emphasize a practical consideration for health food development.

The findings highlight that functional value should not be presumed based solely on the basis of botanical classification or traditional reputation. Comprehensive, species- and origin-specific compositional analysis, together with appropriate safety evaluation, is essential when utilizing citrus peels as raw materials for health food applications. This commentary does not seek to generalize functional properties across citrus species; rather, it provides an evidence-based, practice-oriented illustration of the need for compositional verification.

## Data Availability

The data presented in this study are available upon request from the corresponding author, E.K., due to the inclusion of analysis data from external institutions.

## Abbreviations

The following abbreviations are used in this manuscript:

HPLC	High-Performance Liquid Chromatography
LOD	Limit of Detection
LOQ	Limit of Quantification
RSD	Relative Standard Deviation
